# Extracorporeal Membrane Oxygenation-Impella (ECPELLA) for Sepsis-Induced Cardiogenic Shock: A Case Report on Vascular Access Selection and Its Use Without Systemic Anticoagulation

**DOI:** 10.7759/cureus.79799

**Published:** 2025-02-27

**Authors:** Hibiki Serizawa, Ginga Suzuki, Toshimitsu Kobori, Yoshimi Nakamichi, Mitsuru Honda

**Affiliations:** 1 Critical Care Center, Toho University Omori Medical Center, Tokyo, JPN

**Keywords:** critical care cardiology, ecpella, impella cp, sepsis and shock physiology, septic cardiomyopathy, va-ecmo

## Abstract

Sepsis-induced cardiogenic shock (SICS) often necessitates mechanical circulatory support, but the efficacy of venoarterial extracorporeal membrane oxygenation (VA-ECMO) alone remains uncertain due to its potential to increase afterload and worsen cardiac dysfunction. The combination of VA-ECMO and Impella (ECPELLA) has emerged as a potential strategy to mitigate these effects, but its application in SICS remains poorly documented. This case highlights ECPELLA as a viable strategy to improve hemodynamics and facilitate weaning in refractory SICS, particularly in patients with vascular access limitations. A previously healthy 39-year-old man presented with septic shock secondary to necrotizing soft tissue infection and was diagnosed with streptococcal toxic shock syndrome. Despite aggressive resuscitation, refractory shock persisted, and transthoracic echocardiography revealed a left ventricular ejection fraction of 30%, consistent with SICS. VA-ECMO was initiated via the right femoral vessels. However, circulatory failure progressed, with a loss of pulse pressure and aortic valve opening, necessitating additional left ventricular unloading. Given extensive femoral necrosis, ongoing ECMO cannulation, and severe right upper limb ischemia, the left subclavian artery was chosen for Impella placement. While this decision was primarily dictated by anatomical constraints, subclavian access may offer advantages such as improved patient mobility and reduced risk of limb ischemia. Following implantation, pulse pressure improved, aortic valve function was restored, and hemodynamics stabilized, facilitating successful VA-ECMO and Impella weaning. Due to the high bleeding risk from multiple debridements, systemic anticoagulation was withheld. Thrombosis risk was mitigated through the use of heparin-containing purge fluid and serial echocardiographic assessment of left ventricular function and intracardiac thrombus formation. No thromboembolic events were observed. He underwent multiple surgical interventions, including amputation and skin grafting, before eventual recovery and transfer to rehabilitation. This case demonstrates that ECPELLA may be a viable therapeutic option for SICS, particularly in cases requiring alternative vascular access strategies. Additionally, in high bleeding-risk patients, it may be feasible to manage ECPELLA without systemic anticoagulation. Further investigation is needed to evaluate its impact on hemodynamics, thrombosis risk, and long-term outcomes.

## Introduction

Sepsis-induced cardiogenic shock (SICS) often necessitates mechanical circulatory support; however, the efficacy of venoarterial extracorporeal membrane oxygenation (VA-ECMO) alone remains uncertain due to its potential to increase afterload and exacerbate cardiac dysfunction [1-3]. While some studies suggest that VA-ECMO monotherapy may be beneficial in septic shock, the evidence remains inconclusive [2-4]. Furthermore, a recent large-scale randomized controlled trial failed to demonstrate a survival benefit of VA-ECMO in cardiogenic shock due to myocardial infarction [5,6]. However, whether these findings translate to SICS remains unclear, as the pathophysiology of sepsis-induced myocardial dysfunction differs from ischemic cardiogenic shock. Unlike myocardial infarction, which primarily involves localized ischemia, SICS is characterized by systemic inflammation, mitochondrial dysfunction, and transient myocardial depression. These factors may influence the response to mechanical circulatory support.

One of the major limitations of VA-ECMO is the increase in left ventricular afterload, which can further impair myocardial function [7]. Although afterload elevation is a recognized concern in all forms of cardiogenic shock treated with VA-ECMO, it may be particularly relevant in SICS, where myocardial depression is often transient and reversible. Increased afterload in this setting may prolong myocardial dysfunction and delay recovery. Impella has emerged as a potential solution to mitigate these limitations, leading to the increasing use of VA-ECMO combined with Impella (ECPELLA) to improve hemodynamic stability [4]. Impella provides direct left ventricular unloading, which can counteract ECMO-induced afterload elevation. However, in the context of SICS, where myocardial dysfunction is driven by systemic inflammation rather than ischemia, the adequacy of left ventricular unloading by Impella remains uncertain. While Impella may reduce ventricular wall stress and improve perfusion, its impact on myocardial recovery in sepsis-induced myocardial dysfunction has not been well studied.

Despite the increasing adoption of ECPELLA in other forms of cardiogenic shock, its application in SICS remains poorly documented. Key unanswered questions include optimal patient selection, the timing of device deployment, and appropriate anticoagulation strategies, given the high risk of both thrombosis and bleeding in septic patients. Further investigation is needed to determine whether ECPELLA provides significant clinical benefits in SICS beyond theoretical hemodynamic advantages.

## Case presentation

A previously healthy 39-year-old man presented with left inguinal pain persisting since the previous day and difficulty moving. On arrival, he was alert, with violaceous purpura and blister formation extending from the left inguinal region to the thigh. His blood pressure was undetectable, heart rate 117/min, and lactate level 4.7 mmol/L, indicating profound shock. The results of laboratory tests are presented in Table 1.

**Table 1:** Blood tests of the patient.

Inspection items	Result	Reference value
C-reactive protein (mg/dL)	43.0	0.0–0.2
Sodium (mEq/L)	135	138–145
Potassium (mEq/L)	4.1	3.6–4.8
Albumin (g/dL)	2.6	4.1–5.1
Total bilirubin (mg/dL)	3.1	0.4–1.5
Blood urine nitrogen (mg/dL)	32	8–20
Creatinine (mg/dL)	4.82	0.65–1.07
White blood cells (×10^3^/μL)	16.1	3.3–8.6
Hemoglobin (g/dL)	12.6	13.7–16.8
Platelets (×10^3^/μL)	239	158–348
Activated partial thromboplastin time (sec)	37.1	24.0–34.0
Fibrinogen/fibrin degradation products (μg/mL)	13.9	<5

Within thirty minutes of presentation, necrotizing soft tissue infection was suspected, and an exploratory incision revealed subcutaneous necrosis. Although no obvious portal of bacterial entry was identified, there was no evidence of minor trauma, insect bites, or mucosal breaches. Group A Streptococcus antigen was detected, confirming streptococcal toxic shock syndrome. Empirical meropenem was initiated along with rapid fluid resuscitation and norepinephrine infusion. An emergency debridement was also planned.

Despite aggressive resuscitation, refractory shock persisted. The patient received norepinephrine at 0.3 μg/kg/min. His blood pressure remained undetectable, indicating ongoing circulatory failure. Echocardiography revealed a left ventricular ejection fraction (LVEF) of 30%, consistent with sepsis-induced cardiogenic shock (SICS). Due to profound hemodynamic instability and a high risk of imminent cardiac arrest, VA-ECMO was prioritized as an emergency intervention to restore systemic perfusion and prevent further deterioration rapidly. Given the extensive necrosis in the left inguinal region, the patient was intubated and placed on mechanical ventilation, and VA-ECMO was initiated via the right femoral vessels.

Following ECMO initiation, partial hemodynamic improvement was observed, but continued vasopressor support and fluid resuscitation were required before stabilization was achieved. The patient underwent emergency debridement. The quadriceps fascia and muscle appeared viable during surgery, and only necrotic adipose tissue was excised. After the procedure, he was transferred to the intensive care unit (ICU).

**Figure 1:**
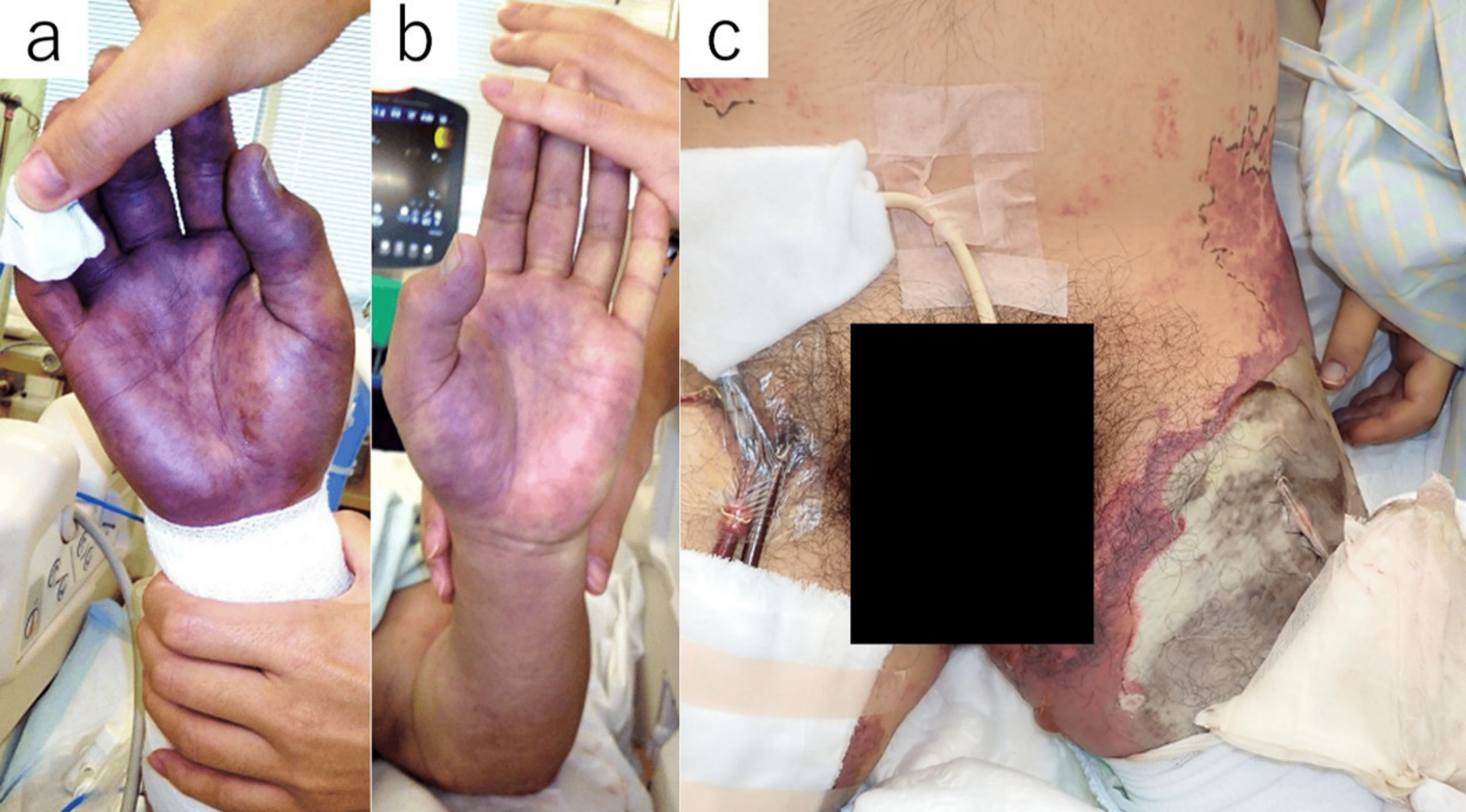
Peripheral perfusion and tissue necrosis in the patient. (a) Significant peripheral circulatory failure in the right hand. (b) The left hand also exhibited peripheral circulatory insufficiency, though less severe than the right hand. (c) Necrosis progression from the left groin to the thigh, resulting in extensive skin and soft tissue necrosis.

Shortly after ICU admission, pulse pressure diminished, and echocardiography revealed further deterioration in left ventricular function, with a loss of aortic valve opening. This suggested worsening myocardial depression. No additional hemodynamic monitoring, such as pulmonary artery catheterization or arterial waveform analysis, was used to guide the decision. Impella insertion was indicated, but vascular access was limited-left femoral artery necrosis, right femoral ECMO cannulation, and severe discoloration of the right hand (Figure 1a). While alternative approaches, including left femoral and right subclavian artery access, were considered, they were deemed unsuitable. The left femoral artery was avoided due to concerns regarding infectious spread and catheter-related infection. The right subclavian artery was not selected because a reduction in distal perfusion could exacerbate skin necrosis, potentially leading to right-hand amputation. Given these constraints, Impella CP SmartAssist (Abiomed, Danvers, Massachusetts, USA) was surgically implanted via the left subclavian artery. Due to concerns about bleeding from open wounds and the need for repeated debridement procedures, systemic anticoagulation was withheld. Alternative anticoagulation strategies were not considered. Thrombus formation was monitored through frequent echocardiographic examinations and local site assessments. No thrombotic or embolic events were observed.

Immediately after Impella implantation, pulse pressure remained absent. However, pulse pressure gradually returned by the following day, and echocardiography demonstrated improved aortic valve opening, suggesting effective left ventricular unloading. The patient’s norepinephrine requirement decreased, and lactate levels began to normalize over the subsequent 24 hours.

By day four, echocardiography demonstrated a significant improvement in LVEF to 60%, allowing for successful ECMO removal. The patient maintained stable hemodynamics with Impella support alone. On day five, norepinephrine was successfully tapered off, and Impella was removed without hemodynamic deterioration. At this stage, circulatory failure had resolved. Although circulatory stabilization was achieved, peripheral necrosis progressed (Figure 1c). Despite circulatory stabilization, peripheral necrosis continued to progress, indicating that irreversible peripheral circulatory failure, rather than systemic circulatory insufficiency, was the primary cause. This necessitated left femoral amputation and additional limb debridement on day 17, which was performed simultaneously with split-thickness skin grafting.

Multiple additional debridement procedures were required due to the formation of nonviable granulation tissue. A tracheostomy was performed on day 25, and the patient was successfully weaned from mechanical ventilation on day 29. After Impella removal, cardiac function remained stable. However, a significant amount of time was required for wound management, physical recovery, and the selection of an appropriate rehabilitation facility. He was transferred to a general ward on day 60 and subsequently to a rehabilitation facility on day 134.

## Discussion

This case highlights three critical aspects: the use of ECPELLA for sepsis-induced cardiogenic shock (SICS), the importance of vascular access selection in a patient with extensive soft tissue necrosis, and the feasibility of managing ECPELLA without systemic anticoagulation in a high bleeding-risk patient.

Reports on the use of ECPELLA in SICS remain scarce [4]. This limited evidence stems from multiple factors, including the unclear benefit of VA-ECMO monotherapy in SICS, uncertainty regarding patient selection for Impella, and the lack of standardized anticoagulation strategies in this population. While observational studies have suggested improved survival with VA-ECMO in SICS [2], subsequent meta-analyses have demonstrated poor outcomes in patients without severe left ventricular depression [3]. These findings indicate that VA-ECMO should be considered in SICS primarily when severe left ventricular dysfunction is present, as extracorporeal support may not provide meaningful benefit in patients with preserved myocardial contractility [2,3].

The poor prognosis associated with VA-ECMO monotherapy in SICS may be due to the increase in left ventricular afterload, which can exacerbate myocardial dysfunction [2,3]. Unlike cardiogenic shock caused by myocardial infarction, where ischemia is the primary insult, SICS involves systemic inflammation, mitochondrial dysfunction, and transient myocardial depression [1]. The addition of Impella to VA-ECMO (ECPELLA) may reduce left ventricular wall stress, improve cardiac output, and prevent further myocardial deterioration [3]. However, further investigation is required to determine whether Impella effectively mitigates the afterload burden imposed by VA-ECMO in SICS.

Timing of left ventricular unloading

In this case, the patient exhibited severe left ventricular depression, justifying the initiation of VA-ECMO. However, shortly after ECMO initiation, pulse pressure decreased, and echocardiography showed a loss of aortic valve opening, indicating worsening myocardial depression rather than inadequate left ventricular unloading. This necessitated the urgent placement of an Impella device. The early recognition of progressive pulse pressure narrowing and aortic valve closure facilitated timely escalation to ECPELLA, which helped prevent left ventricular thrombus formation and worsening pulmonary edema [8].

Although echocardiographic and pulse pressure changes guided decision-making in this case, additional hemodynamic monitoring tools, such as pulmonary artery catheters or left atrial pressure measurements, could provide more precise guidance for Impella initiation [9]. Future studies should evaluate whether invasive monitoring improves patient selection and outcomes.

Vascular access selection and complications

The selection of an appropriate vascular access site was crucial in this case. Alternative options, including the left femoral and right subclavian arteries, were considered but deemed unsuitable. The left femoral artery was avoided due to the risk of infection spread and catheter-related complications. The right subclavian artery was not used due to concerns that impaired distal perfusion could exacerbate skin necrosis and necessitate amputation of the right hand. Given these constraints, Impella was surgically implanted via the left subclavian artery.

While this approach preserved right upper limb perfusion, potential complications such as axillary artery thrombosis or upper limb ischemia should be considered [10]. Routine vascular imaging, including duplex ultrasound or CT angiography, may further optimize access site selection in future cases.

Anticoagulation strategy and risk monitoring

Systemic anticoagulation was withheld due to the high risk of bleeding from repeated debridement and open wounds. While VA-ECMO without anticoagulation has been reported as feasible [9], its safety in ECPELLA patients is unclear. Thrombosis risk was monitored using serial echocardiographic examinations and frequent local site assessments. No thromboembolic events were observed.

Although alternative anticoagulation strategies, such as regional heparinization or low-dose systemic anticoagulation, were not implemented, heparin-containing purge fluid may have provided some degree of thromboprophylaxis. Future studies should evaluate whether inflammatory and coagulation markers, such as D-dimer and fibrinogen, can guide anticoagulation decisions in this setting.

Limitations and future directions

This single-case report limits generalizability, and long-term follow-up data, including cardiac function and vascular complications, are not available. Further research should focus on prospective registries and trials to define optimal patient selection, Impella initiation timing, and SICS anticoagulation strategies.

To address these uncertainties, future studies should evaluate the role of invasive hemodynamic monitoring, advanced vascular imaging for access selection, and standardized anticoagulation protocols in ECPELLA for SICS.

## Conclusions

ECPELLA may represent a promising therapeutic option for patients with sepsis-induced cardiogenic shock (SICS) who exhibit severe left ventricular dysfunction or evidence of increased afterload, such as progressive pulse pressure narrowing or loss of aortic valve opening. In cases of necrotizing soft tissue infection, vascular access selection should be guided by limb perfusion status and the overall surgical strategy. Additionally, in patients at high risk of hemorrhagic complications, ECPELLA may be feasible without systemic anticoagulation, although this approach is not yet standardized and requires careful monitoring for thrombotic complications. Future research should focus on defining optimal anticoagulation strategies and evaluating the long-term safety and efficacy of ECPELLA in this population.
